# The Surfer’s Shoulder: A Systematic Review of Current Literature and Potential Pathophysiological Explanations of Chronic Shoulder Complaints in Wave Surfers

**DOI:** 10.1186/s40798-020-00289-0

**Published:** 2021-01-06

**Authors:** Lisette Charlotte Langenberg, Guilherme Vieira Lima, Sebastiaan Emanuel Heitkamp, Floortje Lutgart Arnoldus Maria Kemps, Matthew Simon Jones, Miguel António de Almeida Garcia Moreira, Denise Eygendaal

**Affiliations:** 1 Department of Orthopaedic Surgery, Amphia, Breda, the Netherlands; 2 Shoulder & Elbow Surgeon, Faculdade de Medicina do ABC e do Hospital Ipiranga, São Paulo, SP Brasil; 3 Fysio Spot/Surffysio, The Hague, the Netherlands; 4 SMC Rijnland Fysiotherapeuten, Leiden, the Netherlands; 5 Trauma and Orthopaedic Registrar, Royal Cornwall Hospital Trust, Treliske, Truro, UK; 6 Faculdade Motricidade Humana, Universidade de Lisboa, Lisbon, Portugal; 7 Amsterdam University Medical Centers, Amsterdam, the Netherlands

**Keywords:** Surfing, Shoulder complaints, Chronic shoulder complaints, Dyskinesis, Muscular imbalance

## Abstract

**Background:**

Wave surfing will debut in the next Olympic Games and is increasingly popular as a sport. Chronic shoulder complaints are frequently reported amongst surfers, though literature researching its pathophysiology and prevention is scarce. This article provides an overview of the current literature, proposes a potential pathogenesis and a potential physiotherapeutic prevention program for surf-induced shoulder complaints.

**Methods:**

A systematic review was performed considering the Preferred Reporting Items for Systematic Reviews and Meta-Analyses (PRISMA) guidelines for articles regarding kinematic analysis of the surf paddling movement. Data sources were Embase, MEDLINE (PubMed) and Research gate. We included case series and cohort studies that originally studied or described the paddle movement made by wave surfers, studies that reported on kinesiologic analysis with nerve conduction studies and studies on 3D motion analysis of the shoulder while paddling.

**Results:**

Eight original articles were included that analysed the shoulder movement in paddling surfers. Muscles that are active during paddling are mainly internal rotators and muscles that are involved in shoulder flexion. Internal rotators are active in propelling the surfer through the water, though external rotator strength is only used while the arm is out of the water.

**Discussion:**

In surfers with shoulder complaints, external rotation range of motion and external rotation strength are impaired. Scapulothoracic dyskinesis may occur and subacromial pain syndrome may coincide. Further research should address potential pectoralis minor shortening, which may lead to aberrant scapular tilt and lateral rotation of the scapula. The surfer’s shoulder is characterised by external rotation deficit, as opposed to internal rotation deficit in the thrower’s shoulder, and it differs substantially from shoulder complaints in swimmers. Therefore, a specific prevention or rehabilitation protocol for surfers is required. Decreased thoracic extension may thereby alter the risk of scapular dyskinesis and hence increase the risk of impingement around the shoulder joint. A potential physiotherapeutic prevention programme should address all these aspects, with the main goal being to increase external rotator strength and to stretch the internal rotators.

**Conclusion:**

There is a high incidence of chronic surf-induced shoulder complaints in surfers. Symptoms may arise due to imbalanced training or scapular dyskinesis, which may subsequently trigger subacromial pain. Physiotherapeutic prevention should include stretching of the internal rotators, external rotator training and optimisation of thoracic extension and scapulothoracic movement.

## Key Points

- Muscles that are active during paddling are mainly internal rotators and muscles that are involved in shoulder flexion.
- In surfers with shoulder complaints, external rotation of the shoulder is impaired. Muscles that are involved in internal rotation of the shoulder may be shortened, leading to aberrant scapular tilt and lateral rotation. Subsequent scapulothoracic dyskinesis may occur and subacromial pain syndrome may coincide.
- Physiotherapeutic prevention should include stretching of the internal rotators, external rotator training and optimisation of thoracic extension and scapulothoracic movement.

## Introduction

The popularity of wave surfing as a sport is increasing. In 2013, it was estimated that there were over 17 million wave surfers worldwide [1]. With a growing number of surfers, surf-related injuries will increase, resulting in a rise in those presenting to medical professionals with their surf-induced injuries and return-to-sport questions. In 2021 wave surfing will be part of the Olympic Games for the first time, which will enhance the medical scientific interest in the sport.

Field studies performed in competitive and recreational surfers showed that the majority of the time (44–61%) in the water is spent paddling, consisting of many repetitive overhead movements [2–9]. Given this, injury due to shoulder overload seems probable. However, research regarding chronic shoulder complaints in surfers is scarce.

The reported incidence of chronic shoulder injuries in surfers in literature is 10–27%. (22.4% of 1348 [10]; 27% of 62 [11]; 18% of 477 [12]; and 10.3% of 136 [13]). Most authors emphasise that these injuries were not caused by a former acute injury. One study reported a high incidence in acute-on-chronic surf-induced shoulder injuries [14]. Impingement is also frequently reported. A questionnaire amongst European physiotherapists revealed a high percentage of shoulder complaints amongst their surfing population. A large amount comprised of chronic shoulder complaints, such as impingement symptoms (Electronic Supplementary Material Appendix S2). In a small case series 23% of the professional surfers that were interviewed reported impingement symptoms, and 31% said to have a tendonitis [15]. Amongst 25 surfers of different levels, 76% had bilateral shoulder complaints and as many as 63% reported impingement symptoms [16]. Overuse injuries of neck, lower back and shoulder are common in recreational surfers [17, 18]. The highest frequency of chronic injuries in surfers is seen in the shoulder and the lumbar spine [10]. A study which measured the shoulder elevation capacity in a group of surf life savers [19] found a significantly negative correlation between hours of board paddling and shoulder elevation. It is thus probable that there is a link between repetitive shoulder exertion during the surf paddling motion and chronic injury in the shoulders of surfers, though research on the potential pathophysiological mechanism is scarce. This highlights a need for structured medical research on this topic.

The questionnaire amongst physiotherapists also showed that none of the respondents that regularly treated surfers used a surf-specific protocol (Electronic Supplementary Material Appendix S2). It would therefore be interesting to compare the surf paddling motion analysis to research regarding overhead sports which are known to cause overuse injury in the shoulder, such as the thrower’s shoulder, glenohumeral internal rotation deficit (GIRD) and shoulder complaints in swimmers; and propose a surf-specific protocol if important differences emerge.

The aim of this review is therefore to evaluate the literature on kinematic analysis of the surf paddling movement; it strives to identify the potential pathogenesis of chronic shoulder complaints in surfers and proposes a physiotherapeutic prevention program.

## Methods

Our research group consisted of an international team of physiotherapists (FK, SH), motion scientists (MM), residents in orthopaedic surgery (LL and MJ) and orthopaedic surgeons (GVL and DE), who all evaluated the included literature from their specific expertise. The group forms the *Surfer’s Shoulder Research Group* of Surfing Medicine International, a non-profit organisation. Via international Skype meetings, agreement was reached within the group regarding terminology and range of motion description. Studies were regarded eligible if original data from case series or cohort studies was published which studied or described the paddle movement made by wave surfers, if a kinesiologic analysis was performed with nerve conduction studies (or electromyography (EMG)) or which included 3D motion analysis of the shoulder while paddling. Kinematic analyses were included that described original data, tested in either a wet or dry lab, with or without wetsuit.

**Fig. 1:**
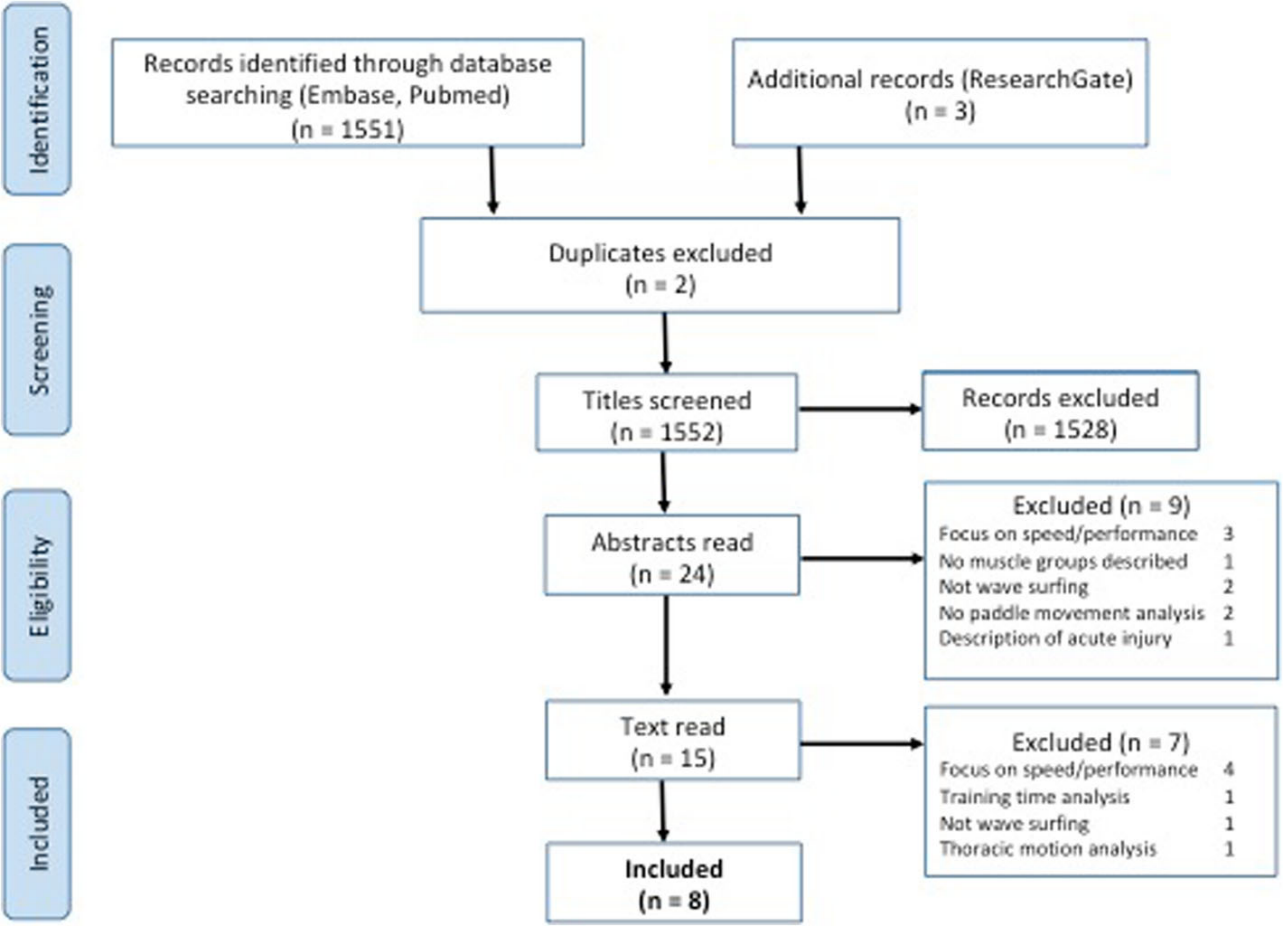
PRISMA flowchart revised

An electronic literature search was conducted in October 2019 using MEDLINE (PubMed) and the Embase databases to identify previous studies describing the different phases of the surf paddle motion or an analysis of the muscles that are active during paddling. The search terms and strategies that were used in PubMed are supplied in the Electronic Supplementary Material as Appendix S1. A systematic strategy following the PRISMA guidelines was used. Figure 1 shows an overview of the search process. “Other resources” include Research gate and personally obtained copies gained at conferences or via email conversations with peers. The search result was screened for eligibility by at least two researchers, LL, FK, SH or GVL, who independently determined whether an article could be included in the review process. If there was doubt regarding eligibility, inclusion was discussed in digital meeting with all authors.

## Results

### Surf Paddling Kinematics

An overview of articles that analysed the surf paddling movement can be found in Table 1.

**Table 1:** An overview of articles that analysed or described surf paddling kinematics

*Study*	*Study type*	*Focus*	*N*	*Findings*
*Surf kinematics*				
Nessler et al. 2015 [20]	Cohort study	Wetsuit vs non-wetsuit effect on muscle activation in paddling; EMG/8 camera Vicon analysis	12	-Mid deltoid, infraspinatus and upper trapezius peaks at recovery phase -Peak triceps brachii, erector spinae and LD during propulsion phase -Mid trapezius activity peak at the end of propulsion.
Nessler et al. 2019 [21]	Cohort study	LD, upper + mid trapezius, post + mid deltoid surface EMG changes at different water velocities	12	-Mid deltoid, post deltoid and trapezius most active at the start of paddle motion -LD is most active in mid-stroke. -LD: propulsion -Deltoid: arm placement and return -Trapezius: scapular rotation
Carter et al. 2015 [19]	Cohort and literature study	Shoulder elevation in relation to paddle activities^a^	54	Negative correlation between hours of board paddling and shoulder elevation scores. Paddle movement (no original data): -Starts in a forward flexed position with scapula protraction, glenohumeral abduction and flexion to catch the water. -Glenohumeral IR and extension follows while the thorax extends. Consistent cycling through these positions predisposes to impingement. -IR is a dominant movement, propelling the surfer, mainly by pectoralis major, LD and subscapularis. -Decreased ER may result as result of subsequent tightness, limiting scapular posterior tilt.
*Posture and ROM*				
Furness et al. 2014 [10]	Retrospective cohort study	Retrospective analysis of chronic injuries in surfers	1348	-Prolonged paddling is leading cause of chronic shoulder injuries. -Shortening of the muscle units of the shoulder muscles may result in muscular imbalance.
McBride and Fisher 2012 [15]	Cross-sectional study	Identification of shoulder complaints in professional surfers	15	Clinical findings in 30 shoulders: -4/30 tendonitis -3/30 impingement. -8/30 had winging of the scapula during shoulder abduction. -2/30 had active signs of external impingement -3/30 had grade I anterior instability
Lassalle et al. 2012 [16]	Cohort study	Physical examination of surfers with and without shoulder complaints	25	76% of surfers had shoulder pain In the painful group: -Impingement in 53–63% -ER decreased compared to the non-painful group
*Shoulder strength in surfing*				
Furness et al. 2018 [22]	Cohort study	Internal to external strength ratio examined in professional surfers and reliability of testing.	21	More strength in IR muscles compared to ER muscles Comparable to other sports with repetitive overhead arm movements
Madeira et al. 2019 [23]	Cohort study	Compares IR in surfers to reference population	5	In surfers: higher values for IR and smaller percentage for ER/IR ratio compared to reference population

*IR* internal rotation, *ER* external rotation, *EMG* electromyography, *ROM* range of motion, *LD* latissimus dorsi muscle

^a^Prone paddling and knee paddling are both mentioned

Only one study described the separate phases of the surf paddling movement in the shoulder [20]. One article mentioned the movement of the shoulder joint while paddling, but it remains unclear whether the author refers to a surfer in the prone position (lying on the board) or knee paddling [19]. Only two articles were found that published original EMG data or 8 Vicon Camera analysis of muscle activity during the surf paddling movement [20, 21]. Many of the articles that did study the surf paddling motion using EMG data did not publish the activity in the separate muscle groups and focused on endurance or performance. A summary of muscle activity that could be extracted from the literature can be found in Table 2.

**Table 2:** Muscle activity during the separate phases of paddling in surfing

Propulsive phase		Recovery phase
Pull phase	Push phase	
		
Thorax extension - Erector spinae Shoulder flexion - Pectoralis major - Triceps brachii	Glenohumeral extension *90° flexion to 10° extension* - Latissimus dorsi - Mid trapezius Internal rotation - Subscapularis	Humeral elevation - Deltoid - Supraspinatus Humeral head stabilisation *prevention of superior translation* - Rotator cuff

The data found in the EMG analysis of the surf paddle motion was consistent with data from front crawl swimming [21]. If the same phases are applied to surf paddling, the paddling movement would also start with a propulsive phase after the hand has entered the water (Table 2). This propulsive phase may be divided into a “pull phase” and a “push phase”.

The main active muscles during propulsive action (pull and push) are the pectoralis major, latissimus dorsi and subscapularis [19, 24, 25]. In the propulsion phase, a peak in activity of triceps brachii, erector spinae and latissimus dorsi were recorded in EMG studies [20, 21]. The humerus mainly internally rotates and flexes in the shoulder joint, while pulling and pushing the body through the water [19]. In surfers, erector spinae provides stability for forceful shoulder flexion and elbow extension [20], the thorax is extended [19, 26] and there is no axial rotation of the spine, contrary to swimming. The primary muscles that now contribute to propulsion of a surfer include mainly the pectoralis major and the triceps brachii [21]. During the push phase, the glenohumeral joint is extending (from 90° flexion to 10° extension), adducting and internally rotating. At the end of the push phase, the mid trapezius shows an EMG activity peak [20].

When the arm is lifted out of the water, the recovery phase starts. In humeral elevation, the deltoid muscle is of main importance [20, 27], followed by the supraspinatus. To prevent the humeral head from superior translation during deltoid contraction, the remaining rotator cuff muscles stabilise the glenohumeral position when the arm is flexed forward [28]. Eight camera Vicon motion analysis of surfers shows middle deltoid, infraspinatus and upper trapezius peaks in the recovery phase [20]. The middle deltoid is most active in the late recovery phase [20].

## Discussion

### Potential Pathophysiological Pathway of Chronic Surf-related Shoulder Complaints

An overview of the potential causes of chronic shoulder complaints in surfers can be found in Table 3.

**Table 3:** Potential pathological pathways of development of chronic surf-induced shoulder complaints

Diagnosis	Pathophysiologic mechanism	Causes	References
**Muscular imbalance/scapulothoracic dyskinesis**	Imbalanced training of external and internal rotators	External rotation only occurs when the arm is out of the water, Pectoralis major, subscapularis and latissimus dorsi well trained in propelling movement of paddling	[16, 19–23]
	Muscle fatigue		[25]
**Impairment of subacromial space predisposing impingement**	Cycling movement of flexed forward and internally rotated shoulder.	Possible anterior glenohumeral displacement. Possible repetitive decrease of subacromial space	[19, 29, 30]
	Forcible elevation at start of pull phase.	Comparable to Neer sign	[31]
	Decreased thoracic extension	Possible repetitive decrease of subacromial space	[10, 16, 31]
**External factors**	Material: wetsuit strain	Impaired ROM, faster cuff fatigue, higher deltoid activity	[20, 27]
	Environmental factors	Water temperature, current/water flow	[4, 21]
	Material: board type	More shoulder complaints in longboarders	[16]
***Further research required; applicable to surfers?***	*Shortening of pectoralis minor?*	*Anterior scapular tilting*	[16, 19, 28, 29, 32, 33]
	*Neuropathy of suprascapular nerve or thoracic longus nerve?*	*Impaired external rotator strength leading to scapular dyskinesis*	[34]

We conclude from our kinematic analysis review that the only moment when the shoulder externally rotates is when the arm is out of the water. Pectoralis major and subscapularis are thus well-trained against resistance, whilst the external rotators are only used against gravity. This complies with a study that hypothesised that an imbalance between internal and external rotators may predispose to muscle tightness and opposed weakness [35] and with two studies that found relatively impaired external rotation strength in wave surfers [22, 23].

In popular surf literature and in surf tutorials, instructors recommend to keep the elbow elevated in the recovery phase [36, 37]. It would be interesting to further research how this slight external rotation in the shoulder joint influences the balance of the surrounding muscles or to compare the technique of surfers with shoulder complaints to those without.

Only one of the studies that were found following the systematic search included the pectoralis minor muscle in their analysis [16]. No significant difference was found between groups of surfers with and without shoulder complaints; however, the conclusion was that the tests used may not be suitable for surfers. The pectoralis minor is known to cause scapula downward rotation, internal rotation and anterior tilt of the scapula [28]. A shortened pectoralis minor muscle may lead to altered scapular movement in shoulder flexion [32]. Patients with a short resting length of the pectoralis minor muscle may have scapular kinematic patterns that are also seen in patients with subacromial pain [28]. This may be explained by increased scapular protraction that leads to narrowing of the subacromial space [29]. Current clinical research by one of the authors suggests a painful triggerpoint at the coracoid process in surfers with shoulder complaints, a study in volleyball players showed this may imply high tension or tendonitis around the pectoralis minor muscle [33]. It would be interesting to test pectoralis minor activity and effects on scapular tilt and subacromial pain in surfers.

Potentially some external or environmental factors are also of influence. Kinematic research showed a significantly higher activity in the deltoid muscle if a 2-mm neoprene wetsuit was worn [20, 27] or when water velocity in a pool was increased [21]. Former research thereby proved that environmental factors when surfing in the ocean are of influence on physical demands [4]. Most kinematic analyses of the surf paddle movement were however performed in a controlled environment, like a swimming pool or a dry laboratory. Environmental or material factors should be included in future research that addresses shoulder complaints in surfers.

The role of muscle fatigue should be researched more thoroughly. Though some literature states against influence of weakness after a certain time of exertion, tested exposure time was short [20, 27]. A study that analysed front crawl swimming showed continuous activity of the serratus anterior and subscapularis, and emphasised that these muscles may thus be susceptible to fatigue [25]. In a study that analysed 78 competitive swimmers without shoulder complaints, scapular dyskinesis was found in 82% following a swim training. The researchers postulated that the muscle imbalance might be due to muscle fatigue and potentially precedes shoulder pain due to the stress on the labrum, anterior capsule and supraspinatus tendon [29]. This may also be true for surfers.

### Potential Explanation for Subacromial Pain

When the humerus is flexed forward and internally rotated, superior and anterior glenohumeral displacement may be noted [30]. By consistently cycling through a paddling movement, the subacromial space may thus be repetitively decreased, which may predispose board paddlers to subacromial pain [19, 29]. Excessive upper trapezius training may also be associated with subacromial pain [28]. This is one of the muscle groups that plays an active role in shoulder abduction, so surfers may be more prone to subacromial pain [25]. The forcible elevation that occurs when the hand is placed in the water thereby yields comparable forces on the muscles surrounding the shoulder as the Neer sign. Hence, repetitive forced shoulder elevation may lead to subacromial pain syndrome [31]. The subacromial space is thereby also narrowed if the trunk does not adequately extend [10].

### The Surfers’ Shoulder Compared to Shoulder Complaints in Overhead Sports

In the case of an abnormal scapula position or movement, a patient is said to have scapular dyskinesis and may result in shoulder pain [38]. Some authors state that scapular dyskinesis will be present in every overhead athlete with shoulder pain [29], because training or muscle fatigue may induce aberrant scapular movement. However, there is no certainty that a subject will develop shoulder complaints if scapular dyskinesis is present [39].

In overhead athletes, this scapular imbalance may be caused by disturbed scapular protraction, or anterior scapular tilt [29, 38]. Scapular protraction is important in shoulder flexion and internal rotation, and is mainly effected by the serratus anterior and the pectoralis minor muscles. In a questionnaire study amongst 422 volleyball players, 60% reported shoulder complaints. An association was found between shoulder pain and asymmetric coracoid tightness or pectoral shortening [33].

Neuropathy of the suprascapular nerve with consequent infraspinatus weakness or atrophy may also be a cause of shoulder complaints in overhead sports [34]. Clinically, infraspinatus atrophy may be seen, resulting in impaired external rotation and excessive scapular protraction. Repetitive traction on the nerve due to extreme shoulder positions, formation of synovial cysts and eccentric infraspinatus contractions may play a role, according to a review including tennis, baseball and volleyball players [34]. It is possible that such neuropathy is also present in surfers.

The condition known as the thrower’s shoulder, that may be present in for example baseball players, gives rise to an internal rotation deficit via several pathologic pathways [40]. Often a superior labrum tear from anterior to posterior (SLAP) or a partial cuff tear is present, that is associated with internal impingement in the shoulder joint. The movement that predisposes these injuries is when the shoulder is in extension, external rotation and abduction, which occurs during the first phase of throwing [41]. If a SLAP lesion is present, a posterior capsule contraction can develop, which may cause additional posterior shoulder pain and a glenohumeral internal rotation deficit (GIRD). Surfers on the contrary mainly proved to have an external rotation deficit [16, 19, 22], and internal rotation strength is increased in surfers [22]. One study addresses potential anterior impingement in surfers if the shoulder is flexed [16]. Future research could focus on clinical or radiological evaluation of capsular reactions or internal impingement in the shoulder joint in surfers.

There are some similarities between the surf paddle movement and the front crawl stroke in swimming. The same propelling and recovery phases may be identified. The position of the arm at the start of a front crawl movement, when the fingers enter the water, is a forced elevation of the arm, like in the Neer sign [31]. This also applies to surfing. In swimmers with unilateral shoulder complaints, the scalene muscles showed a high activation [42]; the same is potentially true for surfers. For shoulder complaints in swimmers, stroke analysis has been proposed as a preventative measure [24]. Potentially, technical analysis of paddling technique may also be beneficial in the prevention of the development of shoulder complaints.

Several large differences between swimmers and paddling surfers should be noted: while swimming the freestyle stroke, a swimmer rotates around a longitudinal axis, with the head held in line with the body. A surfer on the contrary faces forward with the neck and the thorax in extension, with concomitant lumbar lordosis. Both are associated with scapular dyskinesis [43–45].

### Surf-Specific Training and Rehabilitation

In a pilot study that included an interview of 28 physiotherapists, none of them worked with a surf-specific rehabilitation protocol (Electronic Supplementary Material Appendix S2). In literature, suggestions were made to add a structured training program for rotator cuff and upper body strength to the overall training of competitive surfers [46]. Other authors stated that implementing strengthening of opposing muscles that are not utilised during paddling may contribute to better muscle balance and may thus prevent shoulder complaints [35].

**Fig. 2:**
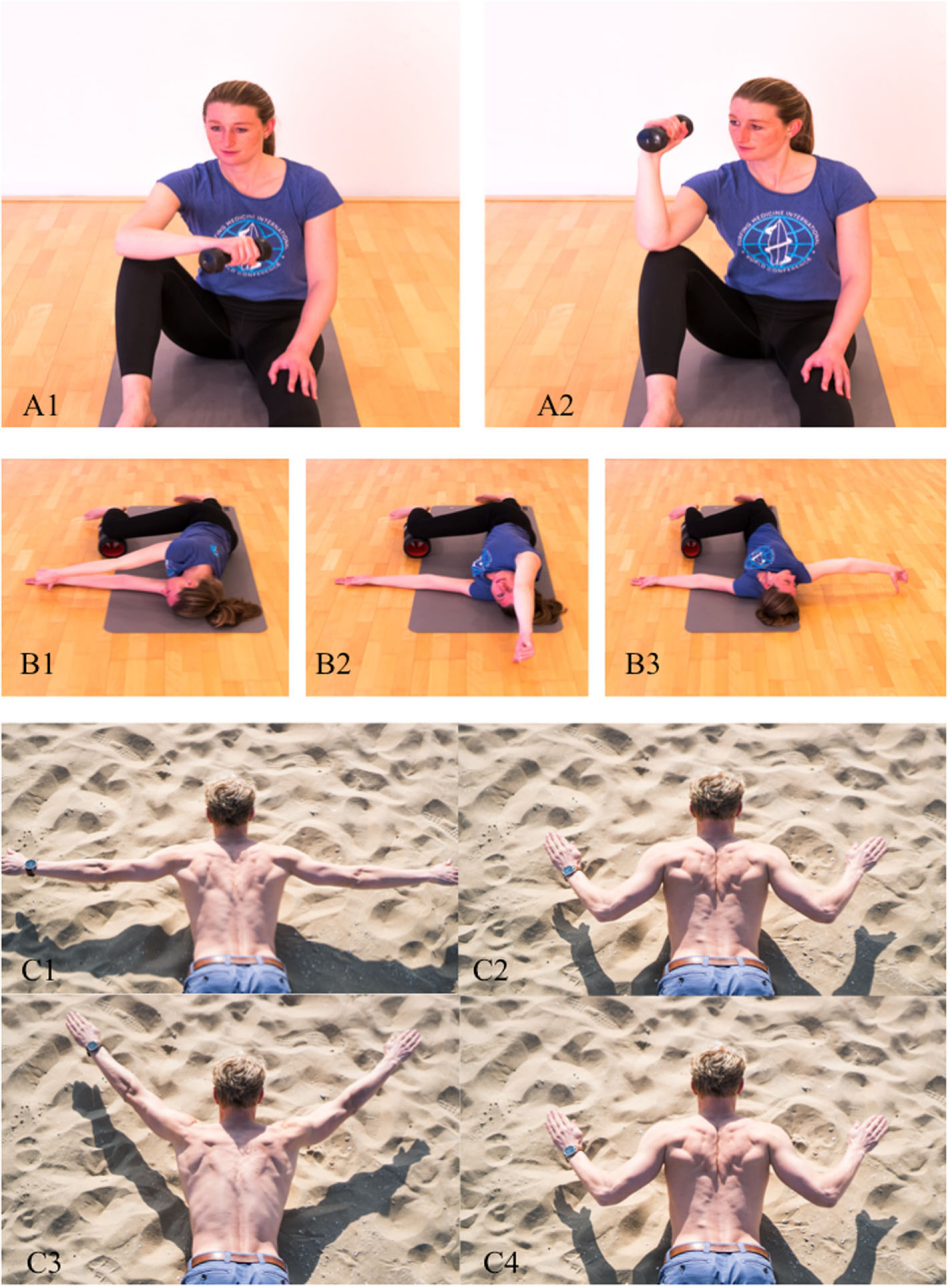
While seated, the elbow rests on the knee (**A1**), the forearm is moved from a horizontal position to a vertical position (**A2**). Purpose: external rotator strengthening. Lying on one side with a straightened leg, the upper leg has the knee flexed in 90° (**B1**). A circular movement is made with the upper arm while attempting to keep the thumb in contact with the floor until 180° of shoulder extension is reached (**B3**). The elbow should remain extended. Purpose: increasing shoulder mobility. While lying down in prone position, slightly extend the lower spine (like the paddle position on a surf board). Starting with the arms in 90° abduction (**C1**), the elbows are pulled down so the arms are in 45° abduction (**C2**). This position may be advanced by adding some external rotation and retroflexion to reach a maximal downward rotation of the scapula and hence enhance scapular posterior tilt and downward rotation. The arms are then brought to 135° abduction (**C3**) and repositioned as in **C2** (**C4**). Purpose: scapular retraction training in combination with external rotator strengthening. All photographic materials are original work from SH, who gave permission for open access publication

**Fig. 3:**
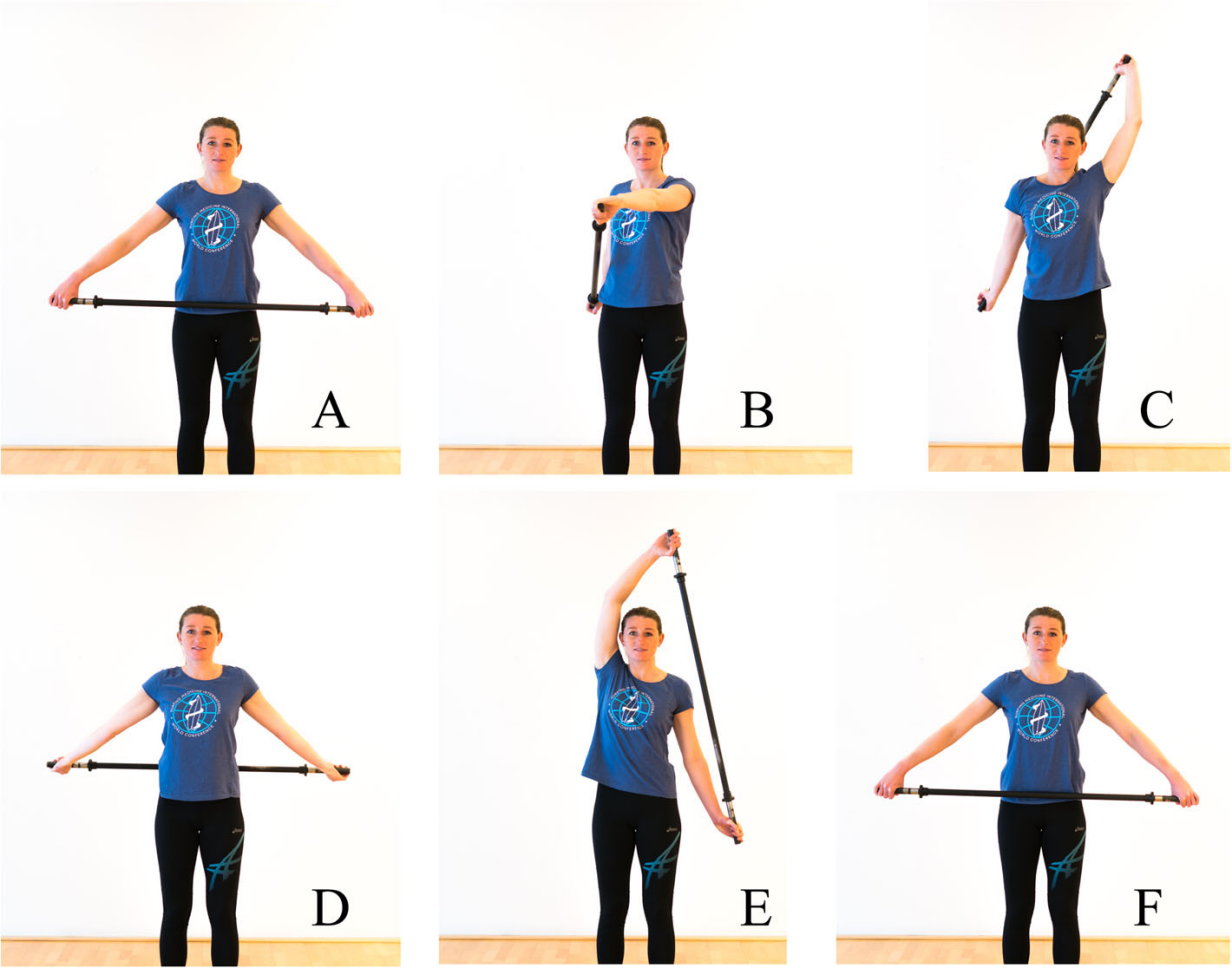
While standing up straight, a stick is held to the thighs with the palms of the hands facing backwards (**A**). One arm is brought up (**B**, **C**) and behind the back (**D**). Then, the other arm is moved up (**E**) and brought forward (**F**). Repeat the exercise starting with the other arm. Purpose: internal rotator stretches. All photographic materials are original work from SH, who gave permission for open access publication

**Fig. 4:**
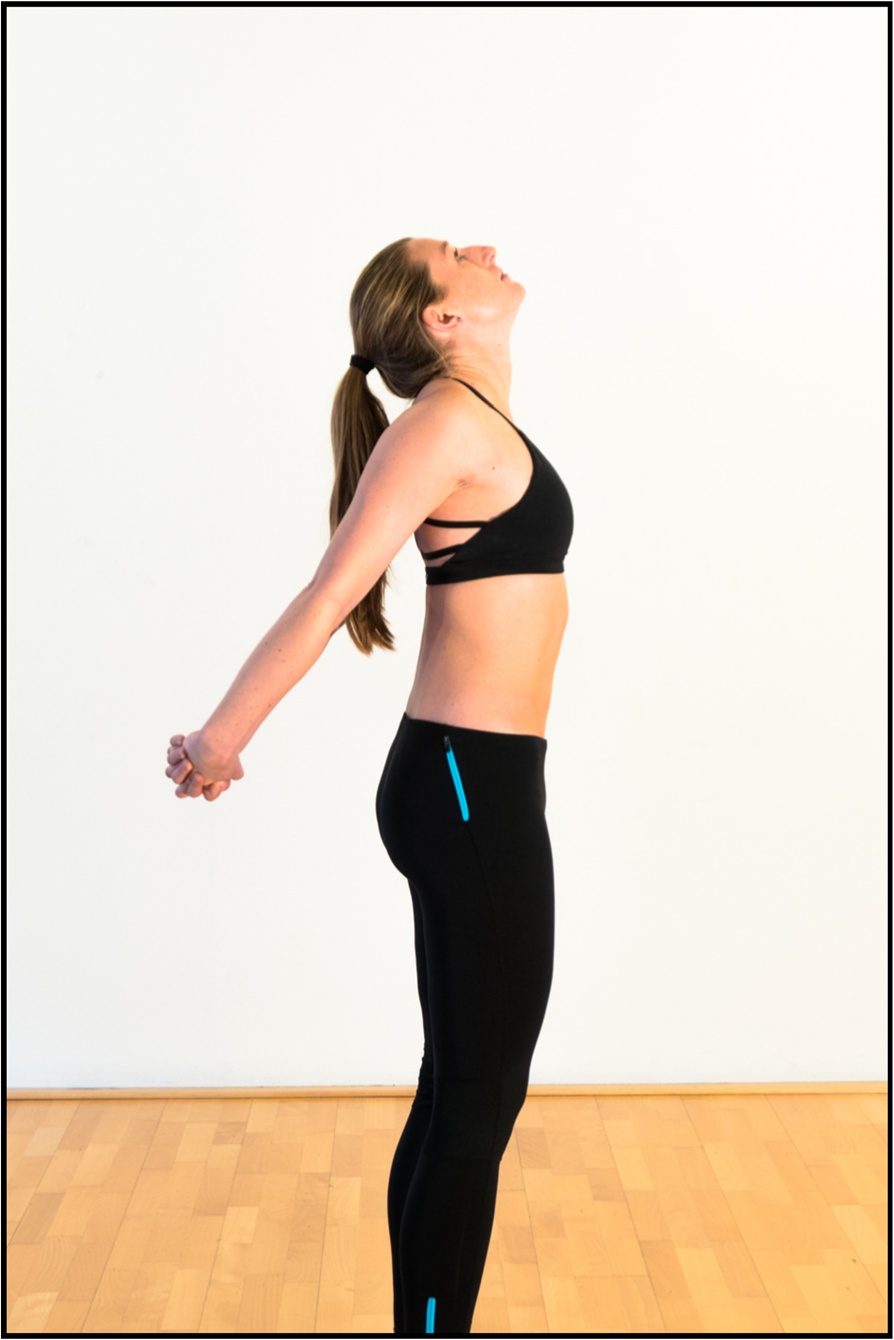
While standing up straight, the hands are folded behind the back. Thoracic extension is performed with retraction of the scapulae. Purpose: stretching pectoralis minor, retracting scapulae. All photographic materials are original work from SH, who gave permission for open access publication

In the case of swimmer’s shoulder, articles have been published that propose an extensive “dry-land” training program [24]. Based on the literature included in this article, we would propose a prevention program for surfers that includes internal rotation stretches and external rotation muscle training. Figure 2 presents an overview of external rotator training exercises, and Figs. 3 and 4 show internal rotator stretch exercises. Analysis of thoracic extension and scapulothoracic movement should thereby be included to prevent subacromial space narrowing.

## Conclusion

Chronic surf-induced shoulder complaints are common, yet poorly analysed. Especially because surfing will debut in the Olympic Games in 2021, we believe that thoroughly performed research on this subject may yield a lot of valuable knowledge. We thereby conclude that the movement made by surfers while paddling, differs from the known pathophysiologic pathways of shoulder complaints in other overhead sports. Further research that focuses solely on wave surfers is therefore necessary.

When systematically analysing the muscle activity in surf paddling, we came to the conclusion that an imbalance seems to exist between the use of internal and external rotators of the shoulder. Pectoralis minor shortening may result, leading to aberrant scapular tilt and lateral rotation, but further research to address the involvement of this specific muscle is needed. Scapulothoracic dyskinesis may occur, potentially aggravated by muscle fatigue, and subacromial pain syndrome may coincide. Decreased thoracic extension may thereby alter the risk of scapular dyskinesis and hence increase the risk of impingement around the shoulder joint. A potential physiotherapeutic prevention programme should address all these features. Neurological impingement, wetsuit strain, thoracic extension and environmental factors may influence shoulder and scapulothoracic movement and therefore enhance the potential for shoulder complaints to occur.

## Supplementary Information

**Additional file 1.** Search string used in PubMed.

**Additional file 2.** Incidence of surf induced shoulder complaints in physiotherapy practices in the Netherlands.

## Data Availability

The search string for this article and the poster of the survey performed by FK and LL that was presented at the annual Surfing Medicine conference in Newquay, Cornwall, UK in 2018, are available as electronic supplementary material (S1 and S2 respectively). The physiotherapeutic exercises are part of the Surfing Medicine International prevention program, that was written by two of the authors (FK and SH, amongst others), it can be accessed via https://www.surfingmed.com/prevention/.

## Abbreviations

EMG: Electromyography
ER: External rotation
GIRD: Glenohumeral internal rotation deficit
IR: Internal rotation
LD: Latissimus dorsi muscle
PRISMA: Preferred Reporting Items for Systematic Reviews and Meta-Analyses
ROM: Range of motion
SLAP: Superior labrum tear from anterior to posterior
