# Supplementary material for: The Surfer’s Shoulder: A Systematic Review of Current Literature and Potential Pathophysiological Explanations of Chronic Shoulder Complaints in Wave Surfers
Source: Sports Med Open. 2021 Jan 6;7:2. doi: 10.1186/s40798-020-00289-0 (PMC7788157; doi:10.1186/s40798-020-00289-0)
Supplement: Supplementary file 1 — Additional file 1. Search string used in PubMed. [file 40798_2020_289_MOESM1_ESM.docx]

Electronic Supplement S1

Search string used in Pubmed

(surf[tiab] OR surfing[tiab] OR surfer*[tiab] OR swimmer*[ti]) AND ("Time and Motion Studies"[Mesh] OR "Athletic Performance"[Mesh] OR "Physical Exertion"[Mesh] OR motion[tiab] OR analys*[tiab] OR kinetic assessment*[tiab] OR countermovement jump*[tiab] OR performance*[tiab])
