# Supplementary material for: The Surfer’s Shoulder: A Systematic Review of Current Literature and Potential Pathophysiological Explanations of Chronic Shoulder Complaints in Wave Surfers
Source: Sports Med Open. 2021 Jan 6;7:2. doi: 10.1186/s40798-020-00289-0 (PMC7788157; doi:10.1186/s40798-020-00289-0)
Supplement: Supplementary file 2 — Additional file 2. Incidence of surf induced shoulder complaints in physiotherapy practices in the Netherlands. [file 40798_2020_289_MOESM2_ESM.pdf]

Incidence of surf induced shoulder complaints in physiotherapy practices in the Netherlands

Floortje A.M. Kemps, PT, MT*, Lisette.C. Langenberg, MD**.

* Fysioholland Amsterdam, the Netherlands
**VU University Medical Center, Amsterdam, The Netherlands

Background

Information regarding surf injuries in the shoulder in the Netherlands is scarce. The surfing population in the Netherlands is growing and to increase the quality of medical treatment, it is important to gain more information on surf induced shoulder complaints.

This pilot study was used to measure the etiology, prevalence and incidence of surf induced shoulder injury in physiotherapy practices in the Netherlands.

Methods

Design

Via social media a general call for information regarding surf related injuries was sent out. All physiotherapists from the Netherlands who were tagged or who responded were sent a short survey.

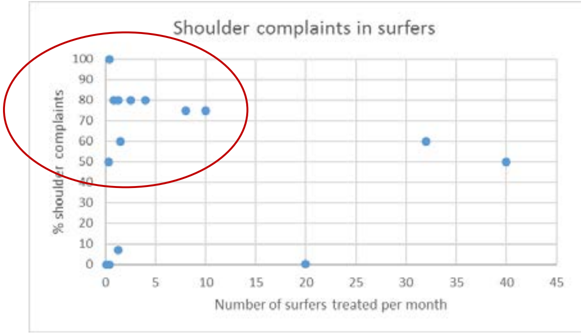

***Table 1**. Number of surfers treated per month and percentage of surf induced shoulder complaints*

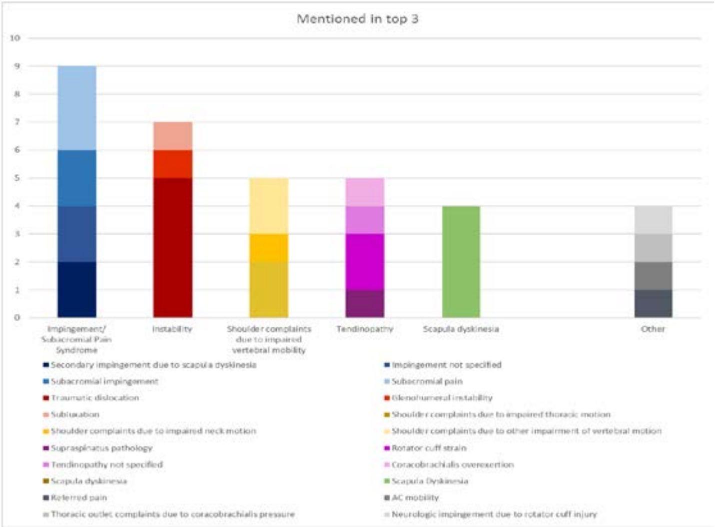

***Table 2**. Top 3 of Surf-induced shoulder complaints reported by Dutch physiotherapists (N=22)*

Results

- Survey was sent to 54 physiotherapists
- Questionnaire regarding the incidence of surf-induced injuries and percentage of shoulder complaints
- Response: 28 physiotherapists (16 practices), of which 19 treated surfing clients
- 50-80% of surfers treated had shoulder complaints
- Top 3 surf induced shoulder injuries:
  1) Impingement / Subacromial Pain Syndrome (SAPS)
  2) Instability
  3) Shoulder complaints due to impaired vertebral mobility (kinematic chain).
- None of the physiotherapists is working with a surf-specific rehabilitation protocol.

Conclusions

There is a broad entry level of surfers in physiotherapy practices in the Netherlands. Amongst these surfers, shoulder injuries seem the most common injury. No protocols are used for rehabilitation.

This pilot study proves that there is a substantial need for further research on the topic of surf induced shoulder complaints and their etiology.
